# Coarticulatory Aspects of the Fluent Speech of French and Italian People Who Stutter Under Altered Auditory Feedback

**DOI:** 10.3389/fpsyg.2020.01745

**Published:** 2020-07-23

**Authors:** Marine Verdurand, Solange Rossato, Claudio Zmarich

**Affiliations:** ^1^Speech Therapy Study, Cabestany, France; ^2^Université Grenoble Alpes, CNRS, Grenoble INP, LIG, Grenoble, France; ^3^Institute of Cognitive Sciences and Technologies, National Research Council, Padua, Italy

**Keywords:** stuttering, coarticulation, acoustic analysis, altered auditory feedback, speech rate

## Abstract

A number of studies have shown that phonetic peculiarities, especially at the coarticulation level, exist in the disfluent as well as in the perceptively fluent speech of people who stutter (PWS). However, results from fluent speech are very disparate and not easily interpretable. Are the coarticulatory features observed in fluent speech of PWS a manifestation of the disorder, or rather a compensation for the disorder itself? The purpose of the present study is to investigate the coarticulatory behavior in the fluent speech of PWS in the attempt to answer the question on its symptomatic or adaptive nature. In order to achieve this, we have studied the speech of 21 adult PWS (10 French and 11 Italian) compared to that of 20 fluent adults (10 French and 10 Italian). The participants had to repeat simple CV syllables in short carrier sentences, where C = /b, d, g/ and V = /a, i, u/. Crucially, this repetition task was performed in order to compare fluent speech coarticulation of PWS to that of PWNS, and to compare the coarticulation of PWS under a condition with normal auditory feedback (NAF) and under a fluency-enhancing condition due to an altered auditory feedback (AAF). This is the first study, to our knowledge, to investigate the coarticulation behavior under AAF. The degree of coarticulation was measured by means of the Locus Equations (LE). The coarticulation degree observed in fluent PWS speech is lower than that of the PWNS, and, more importantly, in AAF condition, PWS coarticulation appears even weaker than in the NAF condition. The results allow to interpret the lower degree of coarticulation found in fluent speech of PWS under NAF condition as a compensation for the disorder, based on the fact that PWS’s coarticulation is weakening in fluency-enhancing conditions, further away from the degree of coarticulation observed in PWNS. Since a lower degree of coarticulation is associated to a greater separation between the places of articulation of the consonant and the vowel, these results are compatible with the hypothesis that larger articulatory movements could be responsible for the stabilization of the PWS speech motor system, increasing the kinesthetic feedback from the effector system. This interpretation shares with a number of relatively recent proposal the idea that stuttering derives from an impaired feedforward (open-loop) control system, which makes PWS rely more heavily on a feedback-based (closed loop) motor control strategy.

## Introduction

Several phonetic studies of stuttered speech have focused on coarticulation, defined as the interweaving between different articulatory gestures associated with different adjacent sounds (Hardcastle and Hewlett, 2006). Among the firsts to explore coarticulation in stuttering, Van Riper (1971, 1982) and Wingate (1969; 1977; 1988; 2002) considered the disfluencies not to be failures in sound production but rather the result of a deficit in the transition between consecutive sounds. This is notably the hypothesis of Wingate’s *fault line*, for which the transition from one phoneme to another within a syllable would constitute a fragile area (*fault line*), on which disfluencies are more likely to occur. The difficulty cannot be with the sound *per se*; the crux of abnormality is the evident failure (better, transient inability) to move on, into the sound that should follow (Wingate, 2002, p. 298–299).

Subsequently, several studies were carried out to test the above hypothesis in adult and children people who stutter (PWS). In the following review, for sake of clarity and synthesis, no results from studies on children will be presented (interested readers could refer to Chang et al., 2002; Subramanian et al., 2003). Most of the studies were made measuring acoustic formants, and particularly second formant (F2) transitions, which are particularly sensitive to changes in tongue advancement during vowel production. Some of these studies showed that F2 formant transitions within disfluent syllables, between the consonant and the following vowel, are either absent or abnormal (for about 85% of the realizations, according to Howell and Vause, 1986; Harrington, 1987).

Coarticulation has also been studied in the perceptually fluent speech of PWS, considering that speech without disfluencies could perhaps already present peculiarities. For this reason, the fluent syllables of the PWS are compared to those of the people who do not stutter (PWNS) using different acoustic measures according to studies: measures of F2, duration of the vowels, area of the vowel triangle and for certain studies also the calculation of the degree of coarticulation using the Locus Equation method (Lindblom, 1963; Krull, 1989). The results of these studies are quite disparate.

Two studies conclude that the articulatory movements are restricted in PWS: in fluent syllables /CV/ and /hVt/ (Klich and May, 1982), and in syllables /CVp/ pronounced both at a normal and fast speech rate (Hirsch, 2007). In addition, this latter author finds, among the persistent PWS, an inadequacy of the speech system to keep pace with rate changes. Indeed, the area of vowel triangle (i.e., the area of the triangle constituted by F2 values for C followed by /a, i, u/ vowels, see Blomgren et al., 1998) of persistent PWS appeared already reduced at normal rate and did not vary with rate increase. On the other hand, by increasing speech rate, PWNS and recovered PWS showed an “undershoot” phenomenon and did not attain the articulatory targets. There was therefore a reduction in the area of the vowel triangle when speech rate increases from normal to fast, suggesting that their speech production system compensated faster speech by making gestures smaller. This adaptation was not found in persistent PWS.

An opposite interpretation is proposed by Robb and Blomgren (1997), whose study focused on the larger and faster tongue movements made by PWS from the closed to the open articulatory position. They measured tongue coarticulation through measurements of F2 formant transitions in a reading task of carrier sentences including /CVt/ syllables (where C = / p, b, s, z/ and V = /a, i, u/). As to plosives, PWS had larger transitions slopes than PWNS. More recently, Dehqan et al. (2016) compared formant transitions in fluent speech segments of Farsi (Persian) PWS and PWNS. Mean overall formant frequency extent was significantly greater for PWS, who also exhibited significantly longer overall F2 transitions. These two studies interpret the larger F2 formant transitions as a manifestation of wider and faster tongue movements.

Others studies rely on the degree of coarticulation as estimated by the slope of the Locus Equation (LE) (Lindblom, 1963; Krull, 1989). An LE describes a 1st order regression fit to a scatter of vowel steady-state frequency values predicting vowel onset frequency values in CV sequences with a fixed C. This measure provides an overall estimation of coarticulation, provided that LE slopes be calculated on CV sequences with vowel pooling and voiced plosives (Tabain, 2000). According to the Hypo and Hyper speech theory (H&H theory, Lindblom, 1990), a weak coarticulation would underlie a non-economic articulatory functioning (large articulatory movements causing more energy expenditure), whereas a strong coarticulation would underlie a thrifty articulatory functioning (more restricted articulatory movements). Whereas Löfqvist (1999) found no support for the slope being an index of the degree of coarticulation, several studies have confirmed the articulatory origins of LE, finding in kinematic domains the same linear relations present in the acoustic domain (Iskarous et al., 2010; Lindblom and Sussman, 2012). Tabain (2000) finds that LEs provide accurate information on the degree of coarticulation for stops, but not for fricatives. This method has already been shown to give information on several types of speech disorders other than stuttering (deafness, dysarthria, and apraxia of speech, etc…see Hardcastle and Tjaden, 2008 for a review). It has been shown to give information on the changes of degree of coarticulation by PWNS when increasing speech rate (LE slopes increase; Berry and Weismer, 2013), when producing prominent syllables (LE slopes decrease; Lindblom et al., 2007) or with more spontaneous speech (LE slopes increase; Duez, 1992).

This method was used by Zmarich and Marchiori (2004) to analyze anticipatory coarticulation (by the vowel on the consonant in a CV syllable) under prosodic stress in four Italian adult PWS and four PWNS. Emphasized/focused syllables are known to be less coarticulated than non emphasized/focused syllables (Lindblom et al., 2007). Results obtained by means of the LE method showed no significant difference between PWS and PWNS. However, the authors noted that, despite the absence of a significant difference, the slope of the LE was higher for PWS than for PWNS on unstressed syllables (higher degree of coarticulation), and lower on stressed syllables under contrastive focus (lower degree of coarticulation). Since the appearance of disfluencies is largely influenced by the word-initial position of the syllable, which also requests, *ceteris paribus*, a lower degree of coarticulation (de Jong et al., 1993; Keating et al., 2004) especially if under contrastive focus (Lindblom et al., 2007), a following study by Pisciotta et al. (2010) carried out the same analysis as Zmarich and Marchiori (2004) but on initial syllables only. The authors found a significantly greater degree of coarticulation (slope of LE) for PWS on initial stressed syllables under contrastive focus only. Sussman et al. (2011) also obtained no significant differences between PWS and PWNS. Results revealed that the LE parameters of both fluent and stuttered syllables were within normal values, although there was a tendency for the most severe PWS to coarticulate less. The authors commented that planning and execution of the anticipatory coarticulation were the same in fluent and disfluent syllables of PWS. The last experimental report is a study by Maruthy et al. (2018), who used a sensitive acoustic technique (spectral coefficient analysis) in order to compare PWS and PWNS with regard to vowel-dependent anticipatory influences as early as the onset burst of a preceding voiceless stop consonant. The observed patterns of anticipatory coarticulation showed no statistically significant differences, nor trends toward such differences, between PWS and PWNS.

To sum up, most studies report no difference between PWS and PWNS (Zmarich and Marchiori, 2004; Sussman et al., 2011; Maruthy et al., 2018), while some found significantly lower coarticulation in PWS (Robb and Blomgren, 1997; Dehqan et al., 2016), and others, on the opposite side, higher coarticulation in PWS (Klich and May, 1982; Pisciotta et al., 2010). These disparate results can be explained by the low number of speakers studied (between 4 and 8, only one study with 10 PWS: Dehqan et al., 2016), the number of different vowels and prosodic conditions, and the number of total occurrences. The speaker-specific variability may be too large to account for coarticulation measured by acoustic measurements, no matter how faithful they are to articulatory movements.

Studies of motor speech control can help interpret the observed data on PWS coarticulation. Namasivayam and van Lieshout (2008) recorded five PWS and five PWNS on a non-word repetition task, /bipa/ and /bapi/. The participants were asked to perform the repetition task at two rates, comfortable and fast, and the movements of the articulators were recorded by means of an electro-magnetic midsagittal articulograph. The results showed that at normal and fast speech rate, PWS exhibited greater upper-lip movement amplitude: the authors assumed that this might reflect a strategy to maintain a stable coordination of movements for the articulatory structure. Namasivayam et al. (2008) continued the investigation by adding the insertion of a bite-block during the repetition task of non-words (/bapi/ and /bipa/). At normal rate, PWS showed a greater range of motion of the upper lip, larger velocity peaks, and longer durations of lip movements compared to PWNS. In contrast, the effect of the bite-block insertion was the same within both groups (larger amplitude and shorter duration of movements, and lower Spatio-Temporal-Index values, see Smith et al., 1995). At rapid speech rate, a significant interaction was found between the groups and the bite-block condition. For PWS, but not for PWNS, the insertion of the bite-block caused an increase in the range of movements and in peak velocity for the lower lip. Again, the authors suggested that PWS make larger movements in order to gain stability. Indeed, the authors of the study relied on the hypothesis of a relationship between range of movement and stability of motor performance (van Lieshout et al., 2004); the larger amplitudes during the insertion of the bite-block could intensify kinesthetic feedbacks and stabilize the movement of speech articulators. Similarly, Namasivayam and van Lieshout (2011) have explained that, in order to speed up the speech rate, two strategies are possible: a reduction both in the amplitude of the movements and in the duration of the segments, allowing the motor system not to increase its speed of operation; or an increase in the speed of movements, allowing the amplitudes to remain unchanged. However, the authors suggested that the first strategy rather leads to a destabilization of the articulatory coordination, since greater variability is found within PWS. More recently, van Lieshout (2017) systematically explored the effects of changes in amplitude (by varying specific segments in a VCV string) and duration (by varying speaking rate) of lips and tongue articulatory gestures and of a measure of the relative phase between the two gestures on sequences of reiterated VCV produced by ten PWNS. The results showed that with small movement amplitudes there was a decrease in coordination stability, independent from movement duration. Thus, these studies on speech motor control show that large amplitude movements could be seen as stabilizers for the speech motor system. In light of this consideration, the low degree of coarticulation found by some studies in PWS would characterize the stable end of the fluency continuum proposed by Peters et al. (2000) and may be interpreted as a sign of compensation for the disorder rather than one of its features.

More recently Didirková and Hirsch (2019) studied coarticulation of articulatory movements for both fluent and disfluent syllables produced by two PWS. They performed a kinematic analysis of the speech gestures involved in the transitions between a stuttered phone and its preceding and subsequent phones by means of electromagnetic articulography (Schönle et al., 1987; Hasegawa-Johnson, 1998). The articulatory configurations were linked to the traditional categories of disfluencies (blocks, repetitions, prolongations, and combined disfluency). The main conclusion was that a stuttering-like disfluency is not always due to a coarticulatory disturbance, since correct coarticulatory patterns of lips, tongue, and jaw can be observed between the disfluent phone and the previous and the subsequent phone. It seems difficult to establish a causal link between disfluency and a low degree of coarticulation. Therefore, the observation of movements greater than usual (and less coarticulated than usual) could be the result of a compensatory strategy to increase stability (Namasivayam and van Lieshout, 2008, 2011) rather than a disturbance of the expected coarticulatory patterns considered as a symptomatic feature of stuttering (Wingate, 2002).

There are, however, other opinions about the larger-than-normal articulatory gestures shown by PWS. Civier et al. (2010) synthesized an acoustic signal by simulating the production of the syllable /bid/ through a neural model of speech production (DIVA, Guenther, 1994). The authors assume that, in tasks requiring speech motor control, PWS are impaired in their capacity to read the feedforward commands (i.e., based on the open loop circuitery) and consequently over-rely on auditory feedback (for a review on sensory feedback and forward modeling, see Desmurget and Grafton, 2000; Perkell, 2012; Guenther and Hickok, 2016; Parrell and Houde, 2019). To prove this hypothesis, the authors biased the DIVA model against feedforward control and towards (auditory) feedback control, resulting in an increase of the frequency of production errors. Indeed, feedback control requires the detection and correction of production errors (e.g., unexpected tongue position or formant pattern). Errors due to reliance on auditory feedback are expected to be the greatest for phonetic events with rapid formant transitions since the rate of acoustic change will exceed the feedback controller’s ability to make timely adjustments. In such a way, the model generated an acoustic signal for [bid] which evidenced the same wide rising of F2 produced by the PWS while producing /bit/ in the experiment of Robb and Blomgren (1997). According to Civier et al. (2010), this wide and fast F2 rising was due to a delayed onset of the F2 transition (caused by the time lag inherent to the auditory feedback) and the acoustic distance between the low F2 locus of the bilabials and the high F2 frequency of the vowels, which cannot be tracked accurately by a feedback-based control system. Therefore, rather than a strategy to increase stability, Civier et al. (2010) hypothesize a weakening of anticipatory, feedfoward command and a greater weighting of auditory feedback in the control of speech production in PWS.

The results described above point to a critical role played by the auditory feedback in stuttering. Indeed, in recent years, many studies have referred to stuttering as a disorder that may present perceptual anomalies (Foundas et al., 2004; Chang et al., 2018) and the role of auditory feedback is particularly intriguing: changing the auditory feedback of PWS can lead to an improvement in fluency (Cherry and Sayers, 1956). The speech of PWS is significantly improved also when they speak under masking noise, Delayed Auditory Feedback (DAF), Frequency Altered Feedback (FAF) or a combination of both (Auditory Altered Feedback AAF), which also helps enhancing fluency in PWS (Howell and Powell, 1987; Kalinowski et al., 1993; Macleod et al., 1995; Natke and Kalveram, 2001; Stuart et al., 2003: Stuart et al., 2004; Armson et al., 2006; Lincoln et al., 2006; Stuart et al., 2008; Antipova et al., 2008; Armson and Kiefte, 2008; Saltuklaroglu et al., 2009). It appears that under the effects of the modified auditory feedback, the rate of disfluencies decreases in most PWS. This improvement varies from one subject to another and depends on the task (Armson et al., 2006; Armson and Kiefte, 2008), the type of alteration (Kalinowski et al., 2000) and the severity of stuttering (Foundas et al., 2013).

Several explanatory hypotheses of the beneficial effect of AAF, not mutually exclusive, have been advanced^1^ : the most relevant of them point to a remediation for inaccuracies in inverse internal models (Max et al., 2004; Daliri and Max, 2018), consisting in a paradoxical normalization of the otherwise limited pre-speech auditory modulation (see below; Daliri and Max, 2018); an involvement of mirror neurons (Kalinowski and Saltuklaroglu, 2003); a neural anchorage to exogenous timing (Etchell et al., 2014).

In particular, the findings from Max et al., summed up in Max and Daliri (2019), potentially bring along important implications, as three relevant phenomena are broadly reported in literature: first, many PWS experience a decrease in the frequency of their stuttering symptoms during consecutive speech in such DAF conditions; second, while the level of pre-speech auditory modulation (consisting in a reduction in amplitude of the electrical activity in planum temporalis when auditory feedback matches auditory expectations) is lower than normal in PWS, it rises under DAF, and it is positively correlated with stuttering frequency during colloquial speech; and third, it has been argued that the fluency-enhancing benefits of DAF are greater for those with more severe stuttering (see Lincoln et al., 2006). This behavioral evidence is supported by a number of neurophysiological researches based on both (1) structural brain imaging that discovered abnormalities in various fronto-parieto-temporal pathways, suggesting that stuttering is associated with deficits in the integration of auditory and motor information for speech production (for a review, see Chang et al., 2018), and (2) animal neurophysiological evidence (Eliades and Wang, 2017; Eliades and Tsunada, 2018). Max and Daliri (2019) concluded that, under typical auditory feedback conditions, adult PWS do not correctly modulate auditory processing prior to the onset of speech, leading to maladaptive, feedback-driven movement corrections that manifest themselves as disfluencies. The speech of the PWS is then disturbed by readjustments that modify the trajectory of the articulators and their coarticulation. This disturbance is weakened with an altered auditory feedback, allowing a more fluent speech as it seems that delayed feedback normalizes the otherwise low pre-speech auditory modulation. The effect of the modified auditory feedback is then assumed to counterbalance the deficiencies of the PWS and allow them to produce more fluent speech, approximating the characteristics of PWNS speech.

While DAF/FAF are known to increase fluency, it is unclear if they impact the coarticulation differences that have been observed in PWS. Consequently, in this experiment, we aim to:

(1) Compare the degree of anticipatory coarticulation of PWS in the so called fluent speech to that of PWNS by means of Locus Equation (LE) in order to ascertain whether it is different from that of PWNS, and, if found different, whether it is higher or lower than that of PWNS;
(2) Use the information gathered in (1) to compare the degree of coarticulation exhibited by PWS under NAF to the degree of coarticulation exhibited by PWS under AAF (which is a condition known to promote a fluency enhancing compensation), in order to obtain insights into the nature, whether symptomatic or compensatory, of the coarticulation in the so called fluent speech of PWS, and on possible articulatory strategies underlying this coarticulation.

Curiously and unfortunately, coarticulation has not been studied, up to our knowledge, for PWS when speaking under AAF. For the study of coarticulation, we used the method based on LE. We suppose LE slope for PWS to be lower than LE slope for PWNS, possibly due to a strategy used to gain in stability (Namasivayam and van Lieshout, 2008, 2011), or to problems in integrating auditory and motor information (Daliri et al., 2017; Daliri and Max, 2018; Max and Daliri, 2019).

As the effect of AAF allows PWS to produce more fluent speech, we postulate that for PWS, were the results on coarticulation measurements under AAF in the same direction of the results obtained for the fluent speech without AAF, the latter could be interpreted as a compensation for the disorder rather than a direct symptom of it. If, as proposed by Max and Daliri (2019), the effect of AAF seems to counterbalance the deficiencies of the PWS, then the consequences on coarticulation should be in the same direction of the values obtained for PWNS.

Furthermore, since most of the previous studies focused on English-speaking populations, and since the degree of coarticulation for a given CV syllable could depend on the language spoken (Sussman et al., 1993; Manuel, 1999), we decided to analyze the coarticulation both in French and Italian PWS and PWNS (French and Italian are both romance languages). The consonants and the vowels constituting the “same” CV stimuli in both languages of the experiment (see below) grossly share the same articulatory features (as for places, manner and voicing; for French, see Léon, 2007; for Italian, see Canepari, 2006; acoustic references for the vowels are, respectively, Gendrot and Adda-Decker, 2005 and Cosi et al., 1995). We also recorded French and Italian PWS when producing more complex syllables CCCV which differed as to their relative frequency in spoken language (Pendeliau-Verdurand, 2014) but this part of the experiment is out of the scope of the present work. Concerning the CV syllables, both Italian and French PWS would be compared to Italian and French PWNS and the results have likely been independent from the language spoken.

We also hold a number of secondary aims, some of which were functional to deepening answers to the two main questions:

(1) Is the hypothesized low degree of anticipatory coarticulation effectively realized by an increase in the amplitude of articulatory gesture? Could this articulatory increase be indexed by the F2 difference between the values at consonant release and at vowel target?
(2) Could derivative measures of ΔF2 and acoustic durations bring some further lighting to the nature of PWS coarticulation? We make reference to the studies measuring the rate of transition (Hz/s), during the whole syllable production or during its beginning by PWS. As a matter of fact, the initial part of a syllable has been discovered to be sensitive to sharp movement accelerations (Civier et al., 2010; Dehqan et al., 2016).
(3) As the last question, we wanted to investigate the possible relationships between stuttering severity and individual difference in coarticulation, as well as in sensitivity to the AAF effects. There are several findings which indicate as the most severe stutterers coarticulate less in fluent speech (Sussman et al., 2011) and benefit more from the application of AAF condition (Max and Daliri, 2019).

## Materials and Methods

### Subjects

In each language, we recorded both PWS and PWNS adults (Verdurand et al., 2013; Pendeliau-Verdurand, 2014). A total of 43 people were recruited, 21 Italian and 22 French. As for French, we recorded 12 PWS recruited through their therapist. Indeed, the first author is a speech and language therapist and recruited the PWSs from among her clients and those of her colleagues. PWNS have been recruited among friends and acquaintances. The recordings took place at the office of the first author (except for one, recorded at home), in a quiet room. As for Italian, 11 PWS were recruited thanks to the Centro Medico di Foniatria in Padova, to Daria Balbo (Speech Therapy student, University of Padova) and to the third author (professor at the same Speech Therapy program), while PWNS were recruited by word of mouth, with the aim to match the subjects for age and sex to PWS. The recordings took place either at the CNR-ISTC, at the CMF or at people’s homes for some PWNS, but a quiet environment was always guaranteed. The samples of French and Italian subjects are not similar as to the males/females ratio, but the same ratio was guaranteed within each language sample.

French and Italian PWS were all diagnosed by speech and language therapists with experience in the assessment and management of stuttering. Based on their answers to a questionnaire, two French PWS were excluded from the study because of associated pathologies; none of the subjects (PWS or PWNS) in the study suffered from hearing impairment, speech/language impairment, or neurological disorders. As for the 11 Italian PWS, all participants had not been in therapy for more than 5 years. As for the 10 French PWS, 6 were into therapy at the time of the experiment, three had not been in therapy for less than 5 years, and one had not been in therapy for more than 5 years. The therapeutic management of stuttering was left to the therapist’s free discretion^2^. This study was carried out in conformity with ethical standards. All the subjects gave informed consent to participate in the study. Table 1 summarizes the speakers analyzed in this study.

**TABLE 1:** Distribution of the number of subjects (mean age in parentheses), according to language and fluency.

	PWNS		PWS	
	Male	Female	Male	Female
French	8 (33)	2 (30)	9 (30)	1 (36)
Italian	4 (33)	6 (46)	5 (33)	6 (28)

### Speech Production Task

The subjects carried out two speech tasks in one session:

– spontaneous speech and reading;
– syllables repetition.

Afterward for PWS, both tasks were performed under altered auditory feedback (AAF, see section “Acoustic Analysis”). The recording of PWNS speech production was limited only to the acoustic signal. That of PWS was both video and audio, in order to disambiguate some particular occurrences of disfluencies like silent blocks, as a function of the assessment of stuttering severity using Stuttering Severity Instrument for Children and Adults-3rd Edition (SSI-3, Riley, 1994), that considers the physical manifestations associated with stuttering.

#### Assessing Severity: Spontaneous Speech and Text-Reading

Spontaneous speech and text-reading tasks allowed the first author to rate the severity of PWS (see Table 2) by means of the SSI-3 (Riley, 1994). Successively, in order to explore the possibility of a relationship between some dimension of the articulatory movements and stuttering severity, we grouped all the subjects according to their stuttering severity assessed by the SSI-3 test and independently from the language (in order to increase statistical power). Because we had missed the raw scores of four French subjects, we used the four-degrees categorical classification of the subjects, and conflated the “very mild” (1 subject) and “mild” PWS (3 subjects) to a unique category (level 1: 4 subjects, 2 French, and 2 Italians). The other two categories were represented by moderate PWS (level 2: 12 subjects, 6 French, and 6 Italians) and severe PWS (level 3: 5 subjects, 2 French, and 3 Italians).

**TABLE 2:** Language, Gender, Age, and Stuttering Severity [according to SSI-3: Very Mild (VM), Mild (M), Moderate (Mo), and Severe (S)] for each PWS.

Participant number	Language	Gender	Age (years)	Severity
S01	French	Male	28	M
S02	French	Female	36	M
S03	French	Male	25	Mo
S04	French	Male	40	Mo
S05	French	Male	27	Mo
S06	French	Male	45	Mo
S07	French	Male	28	Mo
S08	French	Male	32	Mo
S09	French	Male	24	S
S10	French	Male	17	S
S11	Italian	Male	34	VM
S12	Italian	Female	36	M
S13	Italian	Female	24	Mo
S14	Italian	Male	24	M
S15	Italian	Male	46	Mo
S16	Italian	Male	30	Mo
S17	Italian	Female	17	Mo
S18	Italian	Female	22	Mo
S19	Italian	Male	33	S
S20	Italian	Female	25	S
S21	Italian	Female	44	S

#### Syllable Repetition

The French and Italian subjects had to repeat short sentences, immediately after having heard them (see next paragraph), containing the target CV syllables, where C = /b, d, g/ and V = /a, i, u/. For the French subjects, the carrier sentence was ≪*je dis SYLLABE puis SYLLABE puis SYLLABE≫*. For the Italian subjects, the carrier sentence was ≪*Dico SILLABA, poi SILLABA, poi SILLABA≫* The translation is:“*I say SYLLABLE, then SYLLABLE, then SYLLABLE*”for both languages.

The target syllables produced by the 41 subjects were the same in the two languages, and each sentence type was produced three times. The delivery was randomized. We obtained nine occurrences for each syllable type, but because of final F0 declination, the last syllable of the sentences was not considered for the analysis, thus leaving a total of six for each syllable type. Thus, each subject was expected to produce 81 syllables, 54 of which were acoustically analyzed during the repetition task. Therefore, more than 2200 syllables have been analyzed to compare the coarticulation of PWS and PWNS (exactly 1105 for PWNS and 1120 for PWS) and an additional analysis of about 1100 syllables were performed in order to explore the evolution of PWS’s coarticulation under AAF (exactly 1138 syllables).

#### Experimental Procedure

Subjects were sitting in front of the examiner, in a quiet room. The examiner had two computers in front of him. The first computer was equipped with the E-Prime software, which allowed to deliver the stimuli (audio sentences previously recorded by a native speaker), and the passage from one sentence to another was controlled by the examiner. The subject put on the headphones and repeated the sentences. Audio recordings were made using a professional AKG C1000S microphone connected to a Marantz PMD recorder.

PWS participants performed speech tasks in two auditory feedback conditions, in this sequence:

– Normal auditory feedback condition: NAF;
– Altered auditory feedback condition: AAF.

The second computer was equipped with the MaxMSP software (Zicarelli, 1998) for auditory feedback modification. In the AAF condition, the speech of the subject was picked up by the microphone, modified in real time using the software MaxMSP, and redirected to both ears of the subject by means of the earphones. The alteration of the auditory feedback was a combination of a delayed auditory feedback (DAF) with a temporal delay of 60 ms and a frequency shifted auditory feedback with a reduction of 40% of the fundamental frequency (F_0_). This setting was selected on the basis of a pilot study to achieve the maximal fluency enhancement with 4 adults stuttering patients.

**FIGURE 1:**
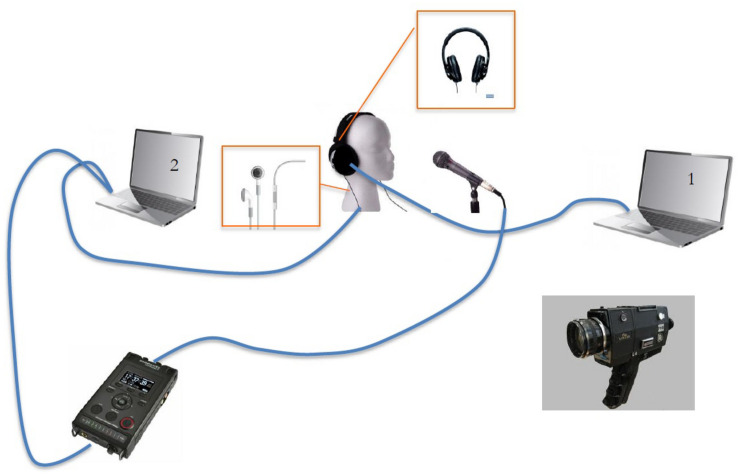
Experimental setting.

Whereas the delay and the lowering of F0 were fixed, the intensity of the AAF was adjusted according to each PWS at a comfortable level and the fluency-enhancing by the AAF was evaluated during the previous tasks of spontaneous speaking and reading and the same level of intensity is kept for the repetition tasks. PWS wore both earphones in which they heard their own speech modified and headphones where they heard the sentences they had to repeat. When disfluencies or errors occurred on CV syllables, the examiner asked the participant to repeat the sentence. Subjects were also video recorded (Figure 1). Thus, in order for a syllable to be targeted for acoustic analysis, the entire phrase needed to be produced fluently and correctly articulated.

### Acoustic Analysis

**FIGURE 2:**
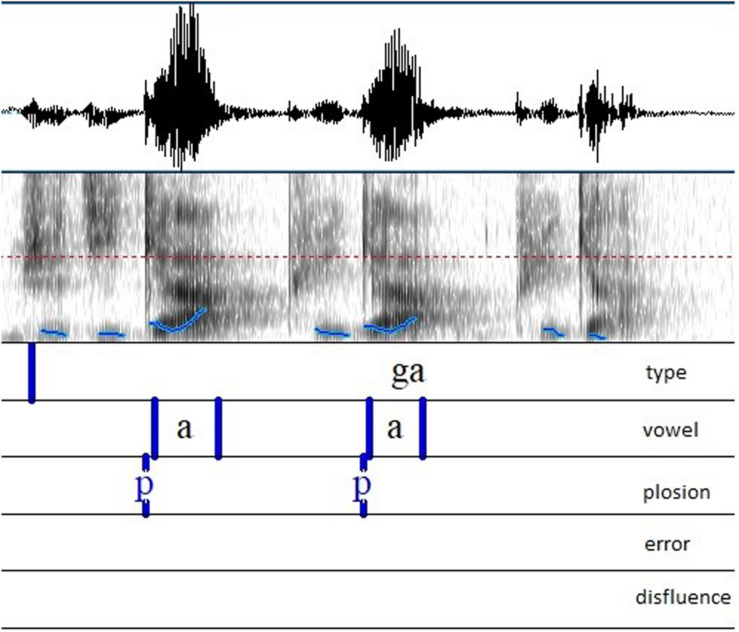
Example of annotation: /ga/.

Using a semi-automatic annotation software, such as EasyAlign (Goldman, 2011), was not possible. Indeed, the disfluent speech, especially in subjects whose stuttering is from severe to very severe, led to too many misalignments. The recording of PWNS could have been processed with EasyAlign (Goldman, 2011), but for the sake of corpus homogeneity we chose to treat all the recordings in the same way. Thus, all the annotations were entirely done manually by the first author using Praat (Boersma and Weenink, 2012). As shown in Figure 2, the annotation includes five tiers. In the first one, named ‘type’, an interval framing the carrier sentence was created. The target syllable was written orthographically. The second tier was used to annotate the vowel. The beginning was placed at the first glottal pulse. The end was determined by the end of the formant structure, particularly F2. In the third tier the release of the plosion was marked. We annotated /p/ (for plosion) if there was a visible/audible release, /f/ (for friction) if we observed/heard a lenition. We also specified the presence of a voicing lead VOT by adding a /v/. Finally, the two following tiers were used to annotate the possible errors of pronunciation and disfluencies. When they occurred during target syllables, theses syllables were not taken into account for the acoustic analysis. Figure 2 illustrates some annotations.

**FIGURE 3:**
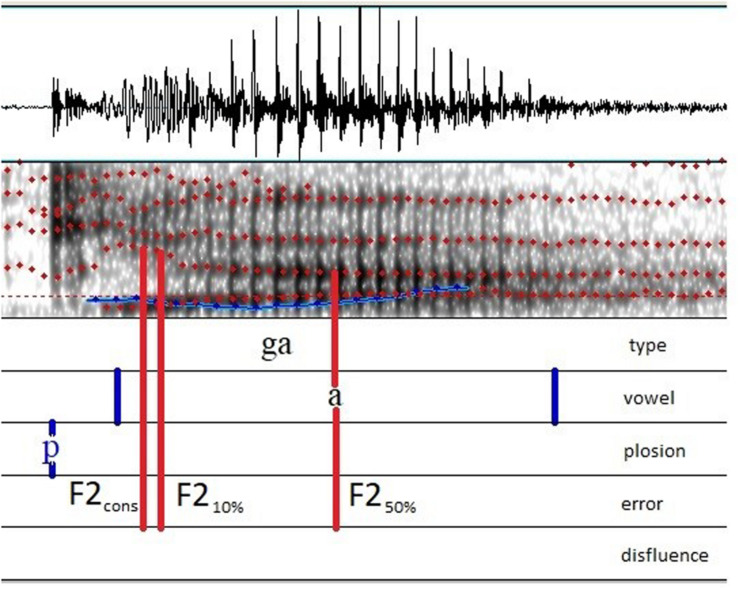
Example of three measurements taken on F2: at vowel onset (F2_cons_), at 10% (F2_10%_), and at 50% (F2_50%_) of the duration of the vowel.

The second formant frequencies (F2) were measured on each CV at three instants as shown in Figure 3:

– at vowel onset, at the beginning of the first clearly recognizable cycles of the vowel (F2_cons_);
– at the first 10 % of the vowel duration (F2_10%_);
– at 50% of the vowel duration (F2_50%_).

F2_cons_ and F2_50%_ allow to quantify the coarticulatory behavior according to the Locus Equation formula (Lindblom, 1963):

$$F\,2_{c\,o\,n\,s}=k*F\,2_{50\%}+b$$

The values of the LE variables were calculated over the 18 occurrences of a given consonant produced in the 3 vowel contexts (/a, i, and u/). Previous research demonstrated the cardinal vowel pooling to approximate well the all-vowel pooling (Berry and Weismer, 2013). The *k* value represents the regression slope that indexes the degree of the anticipatory coarticulation for each plosive consonant. This slope *k* can vary from values near 0 (absence of coarticulation), when the consonant represented by its F2_cons_ is not modified by the following vowel, up to about 1 (high degree of coarticulation), when the realization of the consonant is highly dependent on the following vowel. The *b* value represents the point of intersection of the regression line with the *y*-axis.

In addition, in order to compare the F2 transitions of CV syllables with and without AAF, we quantified the F2 transition for each syllable with the following measures:

– the extent of the whole F2 transition ΔF2 (Hz) defined as the value of F2_50%_ - F2_cons_;
– the rate of the whole F2 transition ΔF2/Δt (Hz/s);
– the initial F2 transition extent, ΔF2_beg_ (Hz), defined as the value of F2_10%_ - F2_cons_;
– the initial F2 transition rate ΔF2_beg_/Δt (Hz/s).

We also measured the duration of the vowels (*duration*, ms). Assuming that subjects could be globally different one from another in terms of coarticulatory habits/capacities, we tried to index this individual characteristic by averaging the values of the considered variable, one by one, across the repetitions of each syllable (ΔF2, ΔF2/Δt, ΔF2_beg_, ΔF2_beg_/Δt, and *duration*). Importantly, here we used the absolute value, and not the positive or negative values resulting from the different combination of any particular C with any particular V. Smaller values in each variable would characterize the syllables produced by subjects more prone to coarticulate and larger values would characterize the syllables produced by subjects less prone to coarticulate. However, we kept the positive or negative values of ΔF2, ΔF2/Δt, ΔF2_beg_, and ΔF2_beg_/Δt when comparing values within each syllable.

## Results

### Results for PWS and PWNS Under NAF Condition

#### Locus Equations

We present the values of *k* from the Locus Equations calculated for the fluent CV syllables produced by PWS and PWNS for both languages. For each participant and each consonant, the slope of the Locus Equation is obtained from 18 pairs of values (F2_cons_ and F2_50%_). Results for the subjects under NAF condition are presented on Table 3 that shows the mean values and standard deviations of *k*.

**TABLE 3:** Mean values and standard deviations (SD) of k for each place of articulation in French and Italian PWNS and PWS.

		Bilabials /b/		Alveolars /d/		Velars /g/	
		*k*	SD	*k*	SD	*k*	SD
French	PWNS	0.85	0.15	0.53	0.12	0.90	0.07
	PWS	0.75	0.11	0.46	0.12	0.83	0.17
Italian	PWNS	0.87	0.04	0.61	0.07	0.95	0.07
	PWS	0.88	0.05	0.59	0.05	0.87	0.07

We carried out a repeated-measure analysis of variance (ANOVA) using R (R Development Core Team, 2017) on the dependent variable *k*, considering the place of articulation of the consonant as a within-subject factor, and the group (PWS/PWNS) and language (Italian/French) as between-subject factors.

Overall, the place of articulation of the consonant strongly influences *k* values [*F*(2,76) = 139.257; *p* < 0.001]. Moreover, the interaction between the place of articulation and the language is not significant, suggesting that the influence of the place of articulation is equivalent in both languages. However, the language spoken has a significant effect on *k* [*F*(1,38) = 18.136; *p* < 0.001]. The value of *k* is lower for French than for Italians for bilabials and alveolars. More importantly, the group category (PWS/PWNS) has significant influence [*F*(1,38) = 9.042; *p* = 0.006] on *k* values, since PWS have lower *k* values than PWNS, without significant interaction with the language spoken. Thus, the differences in *k* values between PWS and PWNS are similar for French and Italian subjects.

#### Derivative Measures of F2 and Duration

Since initial F2 transition extent ΔF2_beg_ (Hz) and rate ΔF2_beg_/Δt (Hz/s) are measures which have proved to critically distinguish PWS from PWNS (Robb and Blomgren, 1997; Civier et al., 2010; Dehqan et al., 2016), we considered them, together with the extent of the whole F2 transition and the rate of the whole F2 transition. All these measures were estimated on each syllable and then we calculated the mean value for each speaker and each syllable across the 6 repetitions of the same syllable, expressed as absolute value. We carried out four separate repeated measure ANOVA, considering the syllable as a within-subject factor, and the group (PWS/PWNS) and the language (Italian/French) as between-subject factors. The absolute extent of the whole F2 transition ΔF2 (Hz) revealed significant differences according to the syllable [*F*(8,310) = 88.836; *p* < 0.001] and the language spoken [*F*(1,37) = 4.717; *p* = 0.036] with a significant interaction between both factors. However, no differences were found regarding the group of the speakers (PWS/PWNS), neither for French speakers nor for Italians (no interaction between group and language factors) in our data. Similar patterns were observed for the other three variables under study, the absolute values of the rate of the whole F2 transition ΔF2/Δt (Hz/s), of the initial F2 transition extent, ΔF2_beg_ (Hz) and of the initial F2 transition rate ΔF2_beg_/Δt (Hz/s), as no significant differences were observed for these variables depending on the group (PWS/PWNS). For the absolute values of ΔF2/Δt, ΔF2_beg_ (Hz), and ΔF2_beg_/Δt, no significant differences were found according to the language spoken, the syllables were the only factor explaining variation of the corresponding variables.

Since PWS speech is often slower than PWNS speech (Bloodstein and Bernstein Ratner, 2008), we also analyzed the duration of vowels with a repeated-measure analysis of variance (ANOVA) on the dependent variable *Duration (s)*, considering the syllable as a within-subject factor, and the group (PWS/PWNS) and the language (Italian/French) as between-subject factors. The type of the CV sequence was a significant factor [*F*(8,319) = 4.371 < 0.001] as it was also the case for the language spoken [*F*(1,38) = 5.350; *p* = 0.026], with a significant interaction between both factors [*F*(8,319) = 4.079; *p* < 0.001]. Indeed, the mean durations of vowel in CV sequences are shorter for Italians than for French speakers (mean = 68.4 ms; sd = 14.4 ms and mean = 77.1 ms; sd = 13.1 ms, respectively) and shorter for the type of CV sequences (/bi/ shorter than /da/). The group category (PWS/PWNS) has no significant influence [*F*(1,38) = 0.020; *p* = 0.887].

### Coarticulation of PWS Under AAF and NAF Conditions

As an effect of the AAF condition, stuttering occurrence virtually reduces to zero for almost all PWS. Table 4 shows the percentages of stuttered syllables out of the total number of syllables constituting the sentences (seven for each sentence, considering also the syllables of the carrier sentence, see section “Syllable Repetition”) under NAF and AAF condition.

**TABLE 4:** Language, Stuttering Severity (according to SSI-3), and measured percentages of stuttered syllables under NAF and AAF condition for each PWS.

Participant number	Language	Severity	Percentages of stuttered syllables (NAF condition)	Percentages of stuttered syllables (AAF condition)
S01	French	M	0.00	4.51
S02	French	M	0.00	0.00
S03	French	Mo	0.00	0.00
S04	French	Mo	1.52	1.48
S05	French	Mo	2.22	0.00
S06	French	Mo	8.09	1.49
S07	French	Mo	8.96	0.00
S08	French	Mo	21.64	0.00
S09	French	S	1.48	0.75
S10	French	S	5.19	0.00
S11	Italian	VM	0.00	0.00
S12	Italian	M	0.00	0.00
S13	Italian	Mo	0.00	0.00
S14	Italian	M	0.00	0.00
S15	Italian	Mo	13.64	0.00
S16	Italian	Mo	0.75	0.00
S17	Italian	Mo	0.00	0.00
S18	Italian	Mo	12.88	0.75
S19	Italian	S	0.77	0.00
S20	Italian	S	11.72	1.57
S21	Italian	S	33.85	0.00

#### Locus Equations

Table 5 shows the mean and the standard deviations of *k* values in each condition (NAF and AAF) for French and Italian PWS.

**TABLE 5:** Values of *k* in PWS, by consonants, language and auditory condition: mean and Standard Deviation.

		Bilabials /b/		Alveolars /d/		Velars /g/	
		*k*	SD	*k*	SD	*k*	SD
French	NAF	0.75	0.11	0.46	0.12	0.83	0.17
	AAF	0.68	0.07	0.32	0.12	0.78	0.20
Italian	NAF	0.88	0.05	0.59	0.05	0.87	0.07
	AAF	0.86	0.03	0.54	0.12	0.89	0.03

**FIGURE 4:**
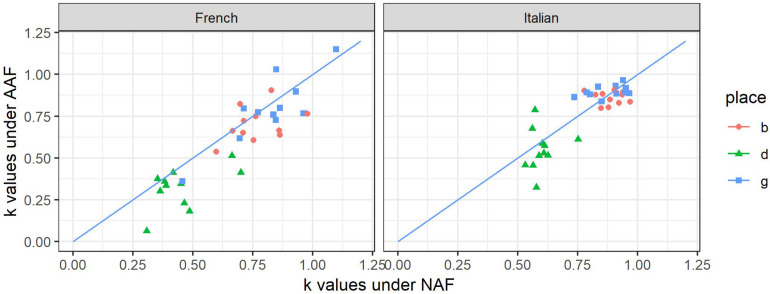
Value of *k* according to the consonants (b, d, g), and groups (PWNS / PWS) for French and Italian subjects under NAF (*x*-axis) and AAF (*y*-axis) condition.

Figure 4 shows the coordinates of *k*_AAF_ as a function of *k*_NAF_ for each French (left) and Italian (right) subject. It can be appreciated the role of the auditory feedback on the values of *k*: if *k* values are higher under AAF, the points will be above the bisector line and vice versa.

We can observe that in French and Italian PWS, AAF condition reduces the value of *k*. Thus, AAF condition further lowers the values previously found for the subjects under NAF conditions. In addition, the values of standard deviations show that inter-individual variability remains high in AAF among PWS of both languages.

We carried out a repeated-measure analysis of variance (ANOVA) on the dependent variable *k*, considering the place of articulation of the consonant and the auditory condition (NAF or AAF) as within-subject factors and the language (Italian/French) as between-subject factor. The statistical results confirm this impression: the auditory condition is significant [*F*(1,100) = 7.966; *p* = 0.006]. PWS have lower *k* values under AAF than in NAF condition. As previously shown, the language has a significant impact on *k* values. However, no significant interaction is found with the auditory condition [*F*(1,100) = 2.970; *p* = 0.088]. Apart from velars among Italian PWS, all *k* values decrease under AAF. So, the trend moves toward less coarticulation under AAF in PWS.

#### Relationship Between Coarticulation Difference and Gesture Amplitude

Assuming that a lower *k* value corresponds to a greater articulatory distance between the consonant target and the vowel target, we considered the absolute value of ΔF2 (Hz) as the acoustic cue of this distance. We verified the relationship between individual *k* values with the individual scores of the absolute value of ΔF2 at each consonantal place mediated across each vocalic context, by correlating them, separately for NAF and AAF conditions and independently from language. We used here the absolute value of ΔF2, and not a positive or negative value depending on the syllables (according to the different combination of C with V). In this way, we obtained a series of individual values where smaller values would characterize the syllables produced by subjects more prone to coarticulate and larger values would characterize the syllables produced by subjects less prone to coarticulate, based on the reasoning that low absolute values of ΔF2 reflect an articulatory proximity of C to V and therefore a stronger coarticulation, and, high absolute values of ΔF2 reflect a relative articulatory distance of C from V and therefore a weaker coarticulation.

In NAF condition, there was a highly significant negative association between *k* values and the mean of the absolute values of ΔF2 for each PWS and each place of articulation (*r* = −0.636, *p* < 0.001 for Pearson product-moment uncorrected scores, based on 63 observations). As for the AAF condition, also in this case there was a highly significant negative association (*r* = −0.663, *p* < 0.001 for Pearson product-moment uncorrected scores, based on 63 observations).

#### Derivative Measures of F2 and Duration

After being reassured about the existence of a trend toward a negative relation between k and the absolute values of ΔF2, we first carried on a repeated-measure analysis of variance (ANOVA) on the absolute values of ΔF2 as a dependent variable, considering the auditory condition (NAF/AAF) and the syllable (/ba, bi, bu, da, di, du, ga, gi, and gu/) as within-subject factors, and the language (Italian/French) as a between-subject factor. The syllable was a significant factor of variation for ΔF2 [*F*(8,388) = 96.784; *p* < 0.001] as expected, but not the language [*F*(1,18) = 0.062; *p* = 0.807]. We found statistical significance for the auditory condition [*F*(1,338) = 8.915; *p* = 0.003], with wider ΔF2 under AAF than under NAF. In addition, we performed two series of separated matched-pairs *t*-test, 9 for each of the two languages, on the ΔF2 mean values of each subject for each syllable under NAF vs. AAF condition (see Table 6).

**TABLE 6:** For each syllable and each language under NAF and AAF condition: Mean values (Hz) of ΔF2, mean of the difference (Hz), 95% of the Confidence Interval (Hz), Standard Deviation of the Differences (Hz), *t* value (Degrees of Freedom), and *p* value (matched pairs *t*-tests).

*Syllables*	*Mean AAF (Hz)*	*Mean NAF (Hz)*	*Mean differ*	*95% C.I.*	*SD of Differ.*	*t(It = 10) t(Fr = 9)*	*p*
***Italian***							
*/ba/*	*73*	*41*	*32*	*2 to 62*	*45*	*2.374*	*0.039*
*/bi/*	131	101	30	−1 to 62	47	2.144	0.058
*/bu/*	−108	−155	47	−3 to 124	115	1.350	0.207
*/da/*	−269	−278	9	−32 to 49	61	0.470	0.648
*/di/*	191	115	76	−26 to 179	153	1.655	0.129
*/du/*	−455	−433	−22	−119 to 75	145	−0504	0.625
*/ga/*	−361	−311	−50	−132 to 32	122	−1.345	0.208
*/gi/*	−4	29	−32	−88 to 23	83	−1.291	0.226
*/gu/*	−129	−125	−4	−59 to 50	81	−0.179	0.861
***French***							
*/ba/*	−77	−78	1	−4 to 33	46	0.068	0.947
*/bi/*	0	−38	38	−39 to 114	107	1.115	0.294
*/bu/*	−*339*	−*157*	−*182*	−*322 to*−*42*	*196*	−*2.939*	*0.017*
*/da/*	−294	−264	−30	−63 to 4	47	−2.003	0.076
*/di/*	31	−8	39	−55 to 133	132	0.934	0.375
*/du/*	−*607*	−*475*	−*132*	−*251 to*−*12*	*167*	−*2.493*	*0.034*
*/ga/*	−*504*	−*442*	−*62*	−*107 to*−*17*	*63*	−*3.116*	*0.012*
*/gi/*	22	6	16	−95 to 127	155	0.328	0.750
*/gu/*	−183	−132	−51	−127 to 25	106	−1.510	0.165

As to Italians, there was only 1 syllable (/ba/) out of 9, where the ΔF2 values for AAF condition were significantly greater than the values of NAF condition, while another one (/bi/) was almost significant. As for French, 3 syllables out of 9 presented values significantly greater for AAF condition than for NAF condition (/bu/, /du/, and /ga/).

We considered also the absolute values of ΔF2/Δt, of ΔF2_beg_, and of ΔF2_beg_/Δt. We carried out three separate repeated measure ANOVA on each of these variables, considering the syllable and the auditory condition (AAF/NAF) as within-subject factors, and the language (Italian/French) as a between-subject factor. Following the observations made with ΔF2, we observed for those three variables that the syllable was a significant factor whereas the language was not. According to the auditory condition, it was significant when comparing the absolute values of ΔF2/Δt [*F*(1,338) = 13.063; *p* < 0.001], when comparing absolute values of ΔF2_beg_ [*F*(1,338) = 32.260; *p* < 0.001] and when comparing the absolute values of ΔF2_beg_/Δt [*F*(1,338) = 5.934; *p* = 0.015].

We performed two series of separated matched-pairs *t*-test, 9 for each of the two languages, on the ΔF2_beg_ mean values of each syllable for each subject under NAF vs. AAF condition (see Table 7).

**TABLE 7:** For each syllable and each language under NAF and AAF condition: Mean values (Hz) of F2 transition extent during the first tenth of the vowel duration (ΔF2_beg_), mean of the difference (Hz), 95% of the Confidence Interval (Hz), Standard Deviation of the Differences (Hz), *t* value (Degrees of Freedom), and *p* value (matched pairs *t*-tests).

*Syllables*	*Mean AAF (Hz)*	*Mean NAF (Hz)*	*Mean differ.(Hz)*	*95% C.I. (Hz)*	*SD of Differ. (Hz)*	*t(It = 10) t(Fr = 9)*	*p*
***Italian***							
*/ba/*	−*40*	−*15*	*26*	*9 to 43*	*25*	*3.344*	*0.007*
*/bi/*	−*57*	−*9*	*48*	*15 to 81*	*49*	*3.211*	*0.009*
*/bu/*	48	42	6	−42 to 55	72	0.293	0.776
*/da/*	35	33	−2	−22 to 17	29	−0.259	0.801
*/di/*	−*58*	*8*	*65*	*23 to 107*	*62*	*3.467*	*0.006*
*/du/*	93	73	−20	−78 to 38	86	−0.772	0.458
*/ga/*	*96*	*34*	−*63*	−*93 to*−*32*	*46*	−*4.567*	*0.001*
*/gi/*	10	3	−7	−28 to 15	32	−0.696	0.503
*/gu/*	54	23	−31	−68 to 7	55	−1.835	0.096
***French***							
*/ba/*	5	7	2	−14 to 18	23	0.272	0.792
*/bi/*	26	−9	−35	−85 to 15	70	−1.598	0.144
*/bu/*	76	8	−67	−143 to 9	107	1.990	0.078
*/da/*	*45*	*26*	−*19*	−*30 to*−*8*	*16*	−*3.802*	*0.004*
*/di/*	−6	−14	−7	−56 to 42	68	−0.338	0.743
*/du/*	*146*	*71*	−*75*	−*108 to*−*42*	*46*	−*5.136*	*0.001*
*/ga/*	*102*	*32*	−*70*	−*98 to*−*43*	*38*	−*5.833*	*0.000*
*/gi/*	−15	16	31	−41 to 104	101	0.980	0.352
*/gu/*	*58*	−*3*	−*61*	−*108 to*−*14*	*66*	−*2.941*	*0.016*

As to Italians, 5 syllables out of 9 exhibited significantly greater values in AAF condition with respect to NAF condition (/ba/, /bi/, /di/, /ga/, and /gi/). As to French 4 syllables out of 9 exhibited significantly greater values in AAF condition with respect to NAF condition (/da/, /du/, /ga/, and /gu/).

Regarding the transition rate ΔF2_beg_/Δt, we performed two series of separated matched-pairs *t*-test, 9 for each of the two languages, on the mean values of each syllable for each subject under NAF vs. AAF condition (see Table 8).

**TABLE 8:** For each syllable and each language under NAF and AAF condition: Mean values (Hz/s) of ΔF2_beg_/Δt, mean of the difference (Hz/s), 95% of the Confidence Interval (Hz/s), Standard Deviation of the Differences (Hz/s), *t* value (Degrees of Freedom), and *p* value (matched pairs *t*-tests).

*Syllables*	*MeanAAF (Hz/s)*	*Mean NAF (Hz/s)*	*Mean differ.(Hz/s)*	*95% C.I.*	*SD of Differ.*	*t(It = 10) t(Fr = 9)*	*p*
***Italian***							
*/ba/*	*3,497*	*1,820*	*1,678*	*677 to 2,678*	*1,490*	*3.736*	*0.004*
*/bi/*	*4,881*	*987*	*3,894*	*1,184 to 6,604*	*4,034*	*3.202*	*0.009*
*/bu/*	−3,859	−6,271	2,411	−2,682 to 7,504	7,581	1.055	0.316
*/da/*	−3,174	−2,717	−457	−2,520 to 1,606	3,071	−0.494	0.632
*/di/*	*5,403*	−*522*	*5,925*	*2,039 to 9,812*	*5,785*	*3.397*	*0.007*
*/du/*	−8,664	−9,255	590	−4,845 to 6,026	8,091	0.242	0.814
*/ga/*	−*8,529*	−*4,305*	−*4,223*	−*6,333 to*−*2,115*	*3,139*	−*4.462*	*0.001*
*/gi/*	−*1,810*	*1,334*	−*3,144*	−*5,586 to*−*702*	*3,635*	−*2.869*	*0.024*
*/gu/*	−4,963	−2,376	−2,587	−6,156 to 982	5,312	−1.615	0.107
***French***							
*/ba/*	−455	−962	507	−1,230 to 2,245	2,429	0.661	0.525
*/bi/*	−2,855	1,314	−4,169	−10,533 to 2,195	8,896	−1.482	0.173
*/bu/*	−8,850	−1,458	−7,391	−17,262 to 2,479	12,841	−1.727	0.122
*/da/*	−3,844	−2,993	−850	−2,176 to 475	1,853	−1.452	0.180
*/di/*	550	1,127	−576	−6,861 to 5,709	8,786	−0.207	0.840
*/du/*	−*13,253*	−*8,449*	−*4,803*	−*8,819 to*−*788*	*5,224*	−*2.758*	*0.025*
*/ga/*	−*9,049*	−*3,438*	−*5,611*	−*9,038 to*−*2,183*	*4,791*	−*3.703*	*0.005*
*/gi/*	1,305	−1,966	3,272	−5,273 to 11,816	11,944	0.866	0.409
*/gu/*	−5,413	443	−5,857	−11,285 to −429	7,588	−2.441	0.037

As to Italians 5 syllables out of 9 exhibited significantly (*p* < 0.05) greater values for AAF condition with respect to NAF condition (/ba/, /bi/, /di/, /ga/, and /gi/). As to French 3 syllables out of 9 exhibited significantly greater values for the AAF condition with respect to the NAF condition (/du/, /ga/, and /gu/).

As a final analysis, we performed a repeated-measure analysis of variance (ANOVA) on the dependent variable *duration* (ms), considering the syllable and the auditory condition (NAF/AAF) as within-subject factors, and the language (Italian/French) as a between-subject factor. Durations of the vowels under NAF appeared to be significantly shorter than durations under AAF (mean = 73 ms; s.d. = 14 for NAF and mean = 102 ms; s.d. = 26 for AAF; [*F*(1,338) = 536.213; *p* < 0.001)]. This vowel lengthening under AAF is similar for French and Italian’s PWS as no significant difference was found for the factor language [*F*(1,18) = 0.707; *p* = 0.411]. We completed our analysis with two series of separated matched-pairs *t*-test, 9 for each of the two languages, on the mean duration values of each syllable for each subject under NAF vs. AAF condition. For both languages, all syllables (9 out of 9) exhibited significantly (*p* < 0.05) higher values for AAF condition with respect to NAF condition.

#### Stuttering Severity

In order to ascertain whether there were any differences among subjects as a function of stuttering severity, we considered the mean value of a parameter averaged across all the values (all syllables) for each PWS under NAF and under AAF. A series of separated repeated model ANOVAs was performed on the mean values for *duration*, the absolute values of ΔF2, ΔF2_beg_/Δt, ΔF2_beg_, and ΔF2_beg_/Δt, and also on *k* (averaged across consonant places), with SSI-3 categories as between factor and auditory condition as within factor (see Table 9).

**TABLE 9:** Mean values for Repeated ANOVAs on dependent variables (duration (ms), absolute values of ΔF2 (Hz), of ΔF2_beg_ (Hz) and of ΔF2_beg_/Δt (Hz/s), and k-slope).

	Between subjects						Within subjects				
		Stuttering severity			*F*	*p*	NAF	AAF	*F*	*p*	Signif. Interact.
		1	2	3							
Duration	NAF	64	77	69	*F*(2,17) = 0.983	0.395	73	103	*F*(1,18) = 51.669	<0.001	NO
(ms)	AAF	106	108	90							
*ΔF2 (Hz)*	NAF	174	200	188	*F*(2,17) = 0.281	0.758	192	223	*F*(1.18) = 12.000	0.003	NO
	AAF	223	229	209							
*ΔF2/Δt*	NAF	5,331	5,275	5,632	*F*(2,17) = 0.672	0.524	5,371	4,489	*F*(1,18) = 9.894	0.006	NO
*(Hz/s)*	AAF	4,348	4,377	4,873							
*ΔF2_beg_*	NAF	26	37	41	*F*(2,17) = 0.222	0.803	36	59	*F*(1.18) = 28.910	<0.001	NO
*(Hz)*	AAF	59	62	49							
*ΔF2_beg_/Δ*	NAF	3,474	4,485	5,158	*F*(2,17) = 0.364	0.700	4,453	5,461	*F*(1,18) = 5.290	0.034	NO
*t (Hz/s)*	AAF	5,163	5,619	5,322							
*k*-slope	NAF	0.738	0.731	0.755	*F*(2,17) = 0.122	0.886	0.738	0.687	*F*(1,18) = 9.344	0.007	NO
	AAF	0.692	0.697	0.661							

*Between subjects variable: PWS degrees of severity (1, Very Mild and Mild; 2, Moderate; and 3, Severe) Within subjects variable: Auditory Condition (NAF and AAF).*

The results of these ANOVAs confirmed the predominant role of auditory condition for all the parameters*:* it was significant for *duration*, the absolute values of ΔF2 were higher under AAF (223 Hz) than under NAF (192 Hz) as well as for ΔF2_beg_, where AAF values (59 Hz) were higher than NAF values (36 Hz) and for the initial transition rate ΔF2_beg_/Δt, with higher values under AAF (5,461 Hz/s) than under NAF (4,453 Hz/s). However, the opposite effect of auditory feedback was observed with the absolute values of ΔF2/Δt: significantly higher NAF values (5,370 Hz/s) than AAF values (4,489 Hz/s) [*F*(1,18) = 9.894; *p* = 0.006]. Therefore, we found for all the parameters except ΔF2/Δt, a tendency to wider F2 extent under AAF. The results suggest larger movements realized with less coarticulation, as confirmed by the statistical significance of the auditory condition on *k*, with lower slope under AAF than under NAF. The severity groups did not differ significantly for any measure. The anticipatory coarticulation degree, as indexed by the different parameters we studied, was not significantly different across the groups with different severity level, and no interaction was found between the severity group and the auditory condition.

As we noted for *k* a difference between the relative order of magnitude for the 3 groups in the two auditory conditions, we performed a series of separated matched-pairs *t*-test, as a function of the severity level. The *k* values under NAF condition were always higher than in the AAF condition: while the difference did not reach statistical significance [*t*_11_ = 1.374; *p* = 0.374] for the very-mild-to-mild subjects (level 1), it was progressively more significant for the moderate subjects (level 2), [*t*_35_ = 1.889; *p* = 0.067] and for the severe subjects (level 3), [*t*_14_ = 3.405; *p* = 0.004].

We recognize the issue of reduced sample size and the unbalanced distribution of stuttering severity across the speakers investigated, as well as the need to be cautious in drawing general conclusions from the analysis that should be considered as avenues for further investigation.

## General Discussion and Conclusion

The primary purpose of the current study was to extend our knowledge on the nature of anticipatory coarticulation, as indexed by the Locus Equation method, in the fluent speech of PWS, first by comparing it to that of PWNS, and second by trying to ascertain whether the resulting differences could be attributed to etiological mechanisms or to compensatory behavior. The secondary purposes were to investigate whether and how the degree of coarticulation and derivative measures of F2 transition and duration could give insights into the way coarticulation behaves in PWS with and without altered auditory feedback.

### Coarticulation of PWS and PWNS

When using the Locus Equation to compare coarticulation effects, PWS (both French and Italian speakers) demonstrate reduced coarticulation compared to fluent controls. While there are coarticulation differences between French and Italian speakers, there are no language by group (PWS vs. PWNS) interaction. Therefore, in this study, the observed values of *k* depend on language (French vs. Italian) and group (PWS vs. PWNS) in addition to the well-known place of articulation of the consonant.

Values of *k* resulting from the Locus Equations are within the range of values obtained in the data of literature, notably those of Sussman et al. (1991, 1993) and Agwuele et al. (2008). In French as in Italian, *k* values are higher for bilabials and velars, and lower for alveolars, the latter being more resistant to coarticulation. The *k* values measured (cf. Table 3) fall within the range of values available in the literature:

– for /b/, between 0.63 (Krull, 1989) and 1.004 (Iskarous et al., 2010);
– for /d/, between 0.25 (Sussman et al., 1993) and 0.59 (Zmarich and Marchiori, 2004);
– for /g/, between 0.71 (Sussman et al., 1991) and 0.97 (Sussman et al., 1998).

These ranges of *k* values are quite wide and some of this variation can be attributed to language. Indeed, Sussman’s studies (1991, 1993) showed that *k* values revealed differences between speakers of English, Urdu, Thai and Arabic. Our results show that even for two languages that are both Romance languages, the language spoken has a significant effect with *k* values lower for French than for Italians for bilabials and alveolars. Therefore, we compared the observed values of *k* with values found in the literature for French and Italian speakers. For French speakers, Duez (1992) obtained mean *k* values across five speakers of 0.75 and 0.81 with C = /b,m/ and of 0.51 and 0.67 for C = /d,n,l/, respectively, for read speech and spontaneous speech (no available data for velars). We obtained slightly higher *k* values with a mean of 0.85 for /b/ and similar values for /d/ (cf. Table 3) for PWNS. For Italian speakers, Zmarich and Marchiori (2004) found the *k* values of the alveolar /d/ averaging 0.59 in four Italian PWNS, and we obtained quite close values with a mean of 0.61 for dentals.

This significant difference in coarticulation as a function of language is hard to explain, because the phonological and the acoustic description of the phonetic system of the two languages do not lead to suspect the existence of differences. As a tentative explanation, we can advance that a difference could reside on phonetic ground: although the consonants are equivalent, vowels could be different. In fact, Mioni (1973) stated /i/ and /u/ to be more closed (higher) in French than in Italian. A further explanation on phonetic ground could rely on different coarticulation strategies within syllables, as it is now widely accepted that the extent and amplitude of the coarticulatory processes often appear to differ among languages when closely examined (Öhman, 1966; Manuel, 1999; Beddor et al., 2002). In addition, in this study, the language factor is found to reach significance when considering *k* values from both groups PWS and PWNS. Further investigations show that, when comparing only PWNS, the *k* values that we obtained for French and for Italian speakers did not reach statistical significance. This result may then be due to reduced statistical power. The observed differences in the degree of coarticulation between Italian and French could also rely on PWS. From this, a second hypothesis has to do with the different treatment history of PWS as a function of the languages: while for all the Italian PWS the last therapy had been administered at least 5 years before, most of the French PWS were under therapy at the moment of the experiment, and possibly some of them were applying some techniques impacting on the coarticulation degree.

More importantly, there are significant differences for *k* values between PWS and PWNS, regardless of their native language as there is no significant interaction in the ANOVA analysis. When considering both languages, PWS present *k* values that were generally lower than those of PWNS. These results are similar to those of Zmarich and Marchiori (2004) who also found, in Italian PWS, a tendency for the focus-accented /dV/ syllables toward lower coarticulation (see also Robb and Blomgren, 1997; Dehqan et al., 2016). From a motor control point of view, this result means that PWS of the present experiment do not tend toward an economy of the articulatory gestures. The articulatory movements implied in the C-to-V sequence are wider, thus resulting in a consonant target, as it is acoustically expressed at the very first beginning of the vowel, that is less influenced by the following vowel target defined by the steady part of the vowel.

The interpretation of lower *k* values in the fluent speech of PWS compared to PWNS remains difficult as it is a global measure, made on several CV sequences, and therefore cannot be clearly attributed to a specific C-to-V sequence and its articulatory gesture. Larger F2 extents and rates were expected in fluent speech of PWS as they reflect a different articulatory strategy compared to PWNS according to the findings about F2 transition differences between PWS and PWNS in previous studies in English speakers (Robb and Blomgren, 1997) and Farsi speakers (Dehqan et al., 2016), and they replicate the results found by Zmarich and Marchiori (2004) on Italians. However, when analyzing the extents and the rates of global or initial F2 transitions, no significant difference was found between the fluent speech of PWS and the fluent speech of PWNS. Indeed, the global measure of *k* reaches statistical significance whereas more local measures derived from F2 transitions did not reach significance. A hypothesis for these apparently inconsistent results may reside in the inherent intra-speaker variability on F2 transitions. This variability was reduced for *k* as each *k* value was estimated over 18 syllables for each place of articulation, whereas ΔF2, ΔF2/Δt, ΔF2_beg_, andΔF2_beg_/Δt have been estimated by averaging over 6 repetitions of the same CV syllable.

It is worth noting that differences in *k* values can not be related to differences in speaking rate as the vowel durations were similar in both PWS’ and PWNS’ fluent speech. Therefore, our findings lead us to the conclusion that these changes in coarticulation are independent from characteristics of individual language or culture as no interaction was found between the Group (PWS and PWNS) and the language (Italian/French), and they may be attributed to some universal neurophysiological mechanisms. By using the words of Dehquan et al., we can interpret that ≪decreased anticipatory coarticulation may result in stuttering speakers applying more lingual adjustment during CV transitions, rather than preparing the tongue for upcoming vowels during production of a preceding consonant≫ (Dehqan et al., 2016: p. 12). The same authors were uncertain about the nature of this behavior, whether compensatory or symptomatic. It may be possible that these late occurring adjustments are symptomatic of altered speech motor planning and/or control. Relating to this, Max and Daliri (2019) excluded an inefficient motor command generation process, pointing instead to a forward modeling generation one.

### Coarticulation of PWS Under AAF and NAF Conditions

Our second main hypothesis was to ascertain whether the resulting differences could be attributed to etiological mechanisms or to compensatory behavior, comparing the two following conditions for PWS: (i) non altered auditory feedback (NAF) and (ii) altered auditory feedback (AAF), that increases fluency for PWS (cf. Table 4), as expected from literature. The main change affecting syllables from NAF to AAF condition consists in a slowing down of the speech rate, as indexed by vowel duration. This result is nothing new, as many studies have reported a slowing down in speech rate when AAF condition is applied (as to PWNS, see Sasisekaran, 2012; Chon et al., 2013; for a review on PWS, see Antipova et al., 2008; Bloodstein and Bernstein Ratner, 2008; Unger et al., 2012). However, we were able to observe some other modifications in different acoustic dimensions. First of all, PWS decrease their degree of the anticipatory coarticulation (as indexed by the *k* value of LE) under AAF condition compared to NAF condition (cf. Table 5). This decrease in coarticulation is loosely associated to an increase in the extent of F2 transition, ΔF2 (Hz), which emerges once that positive and negative intervals are transformed into absolute values, considered as an index of the relative proximity of the consonant and vowel articulations within the same syllable. Indeed, when studying extents and rates of F2 transition, we observed that the auditory condition had a statistical significance on ΔF2, on ΔF2/Δt (Hz/s), on ΔF2_beg_ (Hz) and on ΔF2_beg_/Δt (Hz/s), but the general tendency to less coarticulation under AAF for PWS that appears through the wider rates and extents of the F2 transition is highly dependent on the type of syllable. When comparing the mean values of ΔF2 (Hz) under the two auditory conditions for each syllable, it appears that a few number of syllables, mostly for French, have significantly greater values under AAF condition. When considering ΔF2/Δt (Hz/s), it appears that the global tendency goes to larger values of transition rate under NAF than AAF since the small increase of ΔF2 is counterbalanced with a large increase in vowel duration and, as a result, also in the duration between the beginning of the vowel and its middle. When considering initial F2 transition extent ΔF2_beg_ (Hz) and rate ΔF2_beg_/Δt (Hz/s), almost half of the total number of syllables have greater values under AAF condition than under NAF condition and the initial part of the F2 transition appears to be more affected that the whole transition. Indeed, the absolute mean values of ΔF2_beg_ (Hz) increase from about 38 Hz under NAF to about 56 Hz under AAF (cf. Table 9). In interpreting the results, we emphasize the importance of the F2 formant rate, evidencing how amplitude excursion at the beginning of F2 transition increases proportionally more than duration under AAF with respect to NAF. In other words, amplitude extent is greater under AAF than NAF, meaning greater acceleration in the initial part. Furthermore, if we look at the types of syllables showing the greater increase in F2 extent and rate at vowels’ beginning under AAF condition, we find them mostly to be those vowels requiring steeper formant transitions (Delattre et al., 1955: /bi/, /du/, /ga/, cf. Table 8).

While these results hold for PWS as a group, some intriguing systematic differences emerge when looking at subgroups classified by severity. When the original 5-degrees scale of the SSI-3 is reduced to just 3 degrees, with the two nationalities evenly distributed across the levels, the group of severe PWS apparently stands out and contrasts both the moderate and the mild PWS on some measures. In the passage from NAF to AAF condition, the coarticulation degree decreases. Even though the extent of the reduction is minimal, it is nonetheless enough to bring the syllables of level-3 subjects from the most coarticulated level in NAF condition, to the least coarticulated level in AAF condition. From the results exposed in Table 9, we see that these severe subjects apparently increase the speech rate to a lesser degree, while do not increase significantly the amplitude and the rate of F2 because they are already high from the onset (under NAF condition). However, it should be noted that the small sample size limited a fully clarifying the complex links between the variables in our study, as well as the generalizability of our results. However, our exploratory intent was to test a plausible model of reality able to provide new insights for future research and discussion about the relationship between stuttering severity, coarticulation and sensitivity to AAF effects.

To summarize, the results under AAF condition consist in an increase of both the duration of the vowel in the temporal range and the extent and rate of the initial F2 transition. We believe the increase in initial F2 transition extent ΔF2_beg_ (Hz) and rate ΔF2_beg_/Δt (Hz/s) to be a more critical change than the increase in vowel duration, as it mainly happens in the initial moments of the transition through a fast movement testified by the high value of the formant rate in the first tenth of the vowel duration. This increase could form the articulatory base of the lower-than-normal anticipatory coarticulation exhibited by PWS speech under NAF condition. The associated vowel lengthening found in the speech produced under AAF condition probably contributes to the decrease in coarticulation, by giving more time to PWS to reach the articulatory targets (i.e., less undershoot) with deeper and wider articulatory contacts (Agwuele et al., 2008). The results may be partially consistent with the hypothesis of Civier et al. (2010) that indicates that longer durations and greater frequency transitions that take place in PWS may be due to an over-reliance to control based on auditory feedback. In this hypothesis, phonetic elements that require extensive and fast formant transitions would be slower or longer and more variable in PWS because fast movements are more error-prone when using feedback-based controls (due to time lags in the feedback system). Since a lower degree of coarticulation could result from a greater separation between the places of articulation of the consonant and the vowel, the previous hypothesis is compatible with the hypothesis that larger articulatory movements could be responsible for the stabilization of PWS speech motor system, through an increase of the kinaesthetic feedback from the effector system (see the observation by Agwuele et al., 2008 on deeper and wider articulatory contacts in slow speech). Kalinowski et al. (1993), van Lieshout and Namasivayam (2010), Namasivayam and van Lieshout (2011), and van Lieshout (2017) show that larger movements are stabilizing rather than destabilizing factors in speech. Thus, it is possible, as suggested by Namasivayam and van Lieshout (2008) and van Lieshout et al. (2004), that the modification of the auditory feedback influences the dependence on sensory feedback toward an increased stability of the speech motor control.

All the authors cited in this discussion (and others like Hickok et al., 2011; Tian and Poeppel, 2012; Sares et al., 2018) in the last decade paved the way, with their hypotheses and results, to a shared interpretation of stuttering due to an impaired feedforward (open-loop) control system, which makes PWS rely more heavily on a feedback-based (closed loop) motor control strategy. A potential connection between the results of the present experiment and the role of AAF in stuttering may come from the researches by Max et al. (see for a summary Max and Daliri, 2019), which demonstrated the delayed feedback signal seems to normalize the otherwise low pre-speech auditory modulation (PSAM).

These results also open new clinical perspectives: it could be interesting to propose to PWS to make their articulatory gestures wider, in order to promote a stabilization of their speech motor system and thus, a reduction of disfluencies. At the same time, assessing possible changes in F2 transition behavior subsequent to improved fluency following stuttering therapy may confirm the usefulness of those articulatory maneuvers.

### Limitations

It should be acknowledged that the study described here also have a number of limitations that should be considered, and possibly addressed, in future work. A not remediable flaw is the composite nature of the particular AAF employed. It was a combination of a delayed auditory feedback (DAF) with a temporal delay of 60 ms and a frequency shifted auditory feedback (FAF) with a reduction of 40% of the fundamental frequency (F0). We choose this “formula” because this combination is considered to be the most efficient in establishing fluency in PWS (in our pilot study and in Antipova et al., 2008). The problems with this solution could be that the interpretation of the effects generated by its application would not be uniquely attributable to one of the components rather than to the other.

Another problem is represented by the decision taken at the time of the first author Ph.D. dissertation (Pendeliau-Verdurand, 2014) of not measuring the extent and duration of the “true” F2 transition (i.e., from the beginning of the transition to beginning of the stable portion of the vowel), because not essential in order to establish the degree of anticipatory coarticulation by means of LE method. This absence would be potentially remediable with new segmentations, but requires additional time and resources not available at present.

A further limitation is due to the fact that all the Italian PWS had not been in therapy for more than 5 years, while for French, 6 were still into therapy at the time of the experiment, and only one had not been in therapy for more than 5 years. Adding to this, it was not possible to guarantee that the PWS followed the same kind of therapies for neither the Italians nor the French. This work was initiated in France in the 2010, where the therapeutic management of stuttering was left to the therapist’s free discretion, and the same is true for Italy.

It would be interesting to carry out the same type of analysis on Italian and French-speaking youth which were recorded in order to study the evolution of the degree of coarticulation between young PWS and adult PWS. This kind of comparison is also a well known experimental design to tease apart symptomatic features (already in place from the onset of stuttering in childhood) from compensatory features (emerging later in life). Similarly, the analysis of PWNS under AAF, which we have left aside for the moment, could shed some light on the reduction of coarticulation under AAF of PWS.

In addition, a picture-description task had been recorded for French speakers and it would be interesting to extend this type of recording to Italian speakers and to study this type of speech closer to everyday life than the sentences we have analyzed in this work. In fact, it is unknown how well the present results would correlate to conversational speech or how well they would generalize to words and syllables with different segmental structures.

Suggestions for future regard the investigation of a kind of speech closer to everyday connected speech, under NAF and AAF conditions. As Max and Daliri (2019) stated, if the individual monosyllabic words can be produced by using only feedforward mechanisms, producing complex multisyllabic words and sequences of syllables combined into complete utterances require feedback monitoring and feedback-driven corrections.

## Data Availability Statement

The datasets generated for this study are available on request to the corresponding author.

## Ethics Statement

Ethical review and approval was not required for the study on human participants in accordance with the local legislation and institutional requirements. The patients/participants provided their written informed consent to participate in this study.

## Author Contributions

All authors listed have made a substantial, direct and intellectual contribution to the work, and approved it for publication.

## Conflict of Interest

The authors declare that the research was conducted in the absence of any commercial or financial relationships that could be construed as a potential conflict of interest.

## Footnotes

1 Recently, research focused on the influence of auditory feedback on stuttering has changed in favor of measuring and interpreting speech modifications consequent to sudden and unpredictable perturbations of specific aspects of AF, such as F0 (Sares et al., 2018) or the spatial/timing properties of F1 and F2 peaks for speech targets (Cai et al., 2012, 2014).

2 This was true in the 2010’s in France; currently the vast majority of speech and language therapists are trained in the Camperdown program (O’Brian et al., 2003).
